# Long-term outcome of lymph vessel transplantation after chronic lymphorrhea

**DOI:** 10.1080/23320885.2021.1999245

**Published:** 2021-11-09

**Authors:** Cecilia Dahlbäck, Rüdiger Baumeister, Magnus Åberg, Björn Arnljots, Lieselotte Frost Arner, Håkan Brorson

**Affiliations:** a Department of Clinical Sciences, Lund University, Plastic and Reconstructive Surgery, Skåne University Hospital, Malmö, Sweden; b Department of Micro-, Hand and Reconstructive Surgery, Ludwig-Maximilians-University of Munich, München, Germany

**Keywords:** Lymph, fistula, lymph vessel transplantation, transplantation, lymphedema, lymph node

## Abstract

A 52-year-old male patient developed a chronic fistula with excessive lymph leakage in the left axilla following removal of an enlarged lymph node with chronic local adipose tissue inflammation due to infection. After multiple surgeries, treatment with lymphatic vessel transplantation was successful. No recurrence occurred over 20 years of follow-up.

## Introduction

Within the extremities, the production of lymph (i.e. the ‘lymphatic load’) and transport of lymph (i.e. the ‘lymphatic transport capacity’, that is the amount of functioning lymphatic vessels and lymph nodes) must be in balance [1,2]. Thus, the transport capacity must be higher than the lymphatic load to prevent interstitial accumulation of lymph.

In cases of chronic lymphorrhea, there must be an explanation for the continuous leakage of lymph. Interventions in areas, where the lymphatic transport system is narrowed, such as in the axilla or groin, may to a great extent reduce the lymphatic transport in these areas, which leads to increased pressure within the lymphatic vascular system. In most cases, lymphedema will result clinically. If, however, lymphatic collectors stay open a persistent lymphorrhea with fistulization may result.

Thus, the attempt of a simple closure of the fistula will either induce lymphedema or the fistula will re-open again.

When there is a regional interruption of the lymphatic pathway, lymphatic transplants can successfully improve the lymphatic transport, thus offering an optimal treatment [3–5].

Normalization of the lymphatic transport capacity is possible and has been verified with lymphoscintigraphy [6].

In lymphatic transplantation, viable lymphatic collectors are harvested from the thigh of the patient. Attention is paid to the direction of the lymph flow when the collectors are interposed within the subcutaneous tissue between the upper arm and neck. In the upper arm, they are anastomosed with ascending lymphatic collectors and at the neck also with lymphatic vessels or with lymph nodes [3–5,7–10].

With the help of these natural bypasses, the lymphatic transport is improved, the pressure within the lymphatic collectors, peripheral to the fistula, is reduced. The development of lymphedema is prevented, thus allowing the fistula to occlude.

## Case report

We present a 24-year follow-up of successful lymphatic vessel transplantation performed at the Department of Plastic and Reconstructive Surgery, Skåne University Hospital, Malmö, Sweden in January 1997.

The patient was a married 53-year-old male. He was a heavy smoker and suffered from diabetes. The insulin treatment was hard to regulate despite rigorous controls, and he was thus prone to infections.

The patient sought care at his local hospital in January 1994 with a minor wound on his left index finger. Gradually, he developed bursitis at the elbow, then shortly thereafter, swelling and tenderness in the left axilla. Ultrasound showed a fluid-filled cavity and an incision was made, but very little fluid was evacuated. His fever rose despite antibiotic treatment, and the swelling of the axilla increased. The general surgeon decided to re-operate because the patient had signs of brachial plexopathy. A 100-ml hematoma was evacuated. At the same time, one swollen lymph node was excised because there was no explanation for the hematoma. Pathological examination showed a benign enlarged lymph node. In April lymphoma was suspected due to a palpable mass in the axilla. The tumor was excised and the pathology report showed a non-specific inflammatory reaction and focal fat necrosis but no lymphatic tissue. Computer tomography showed unspecified swelling of the tissues around the axillary artery. No aneurysm was detected. Borrelia serology was negative.

The axillary area was drained multiple times at the patient’s local hospital, with excision of a pseudo capsule three times, diathermy of the lymphatic vessels and injection of tissue glue (Tisseel, 2016 Baxter Healthcare^®^) twice. The pathology report from one of the excisions of the pseudocapsule showed an abscess with heavily dilated lymph vessels. These treatments failed and the patient had to use colostomy dressings to collect the leaking lymph, which amounted to approximately 0.6 L daily. A 10 cm × 7 cm cavity with a 10-cm-long infraclavicular fistula formed. The patient had a limited range of motion when elevating and abducting the arm.

**Figure 1.**
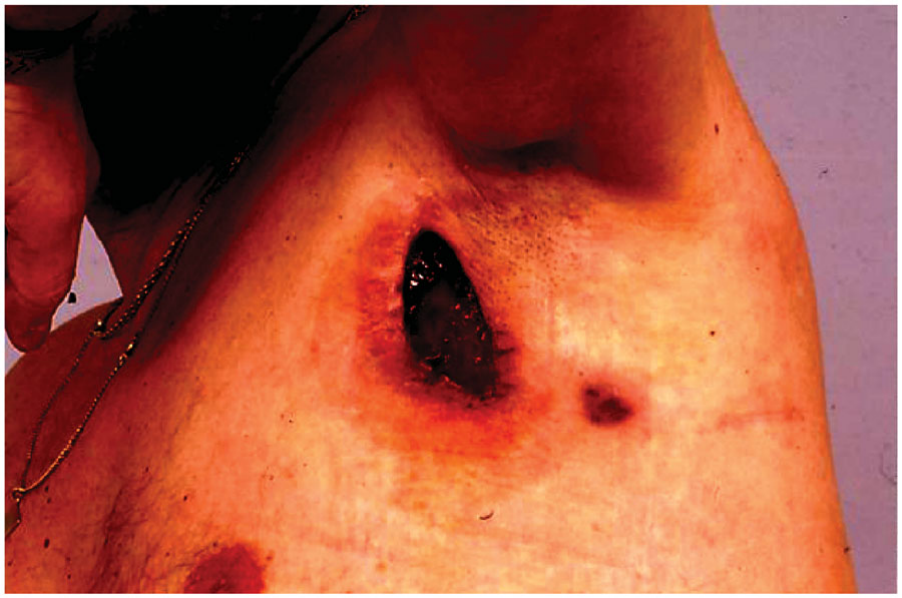
The patient had a 10 cm × 5 cm cavity with a 10-cm-long fistula into the axilla when referred to our department.

Following the surgeries, the patient had a problem with large amounts of lymph leakage from the axilla through a fistula (Figure 1). In October, the patient was referred to our department He never developed lymphedema because all lymph from the arm drained through the fistula.

**Figure 2.**
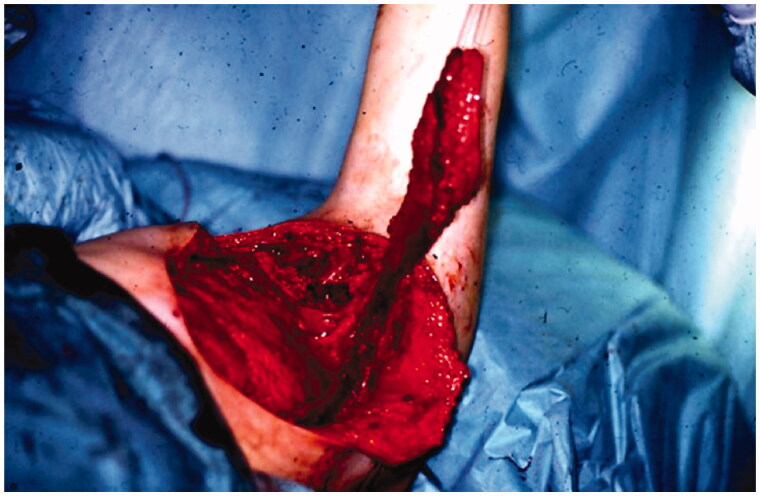
A LD-flap was raised to cover the region of the excised fistula.

In December 1994, a pedicled latissimus dorsi myocutaneous flap (LD-flap) was raised to cover the defect after exploration of the axilla and excision of a pseudocapsule and surrounding fatty tissue (Figure 2).

**Figure 3.**
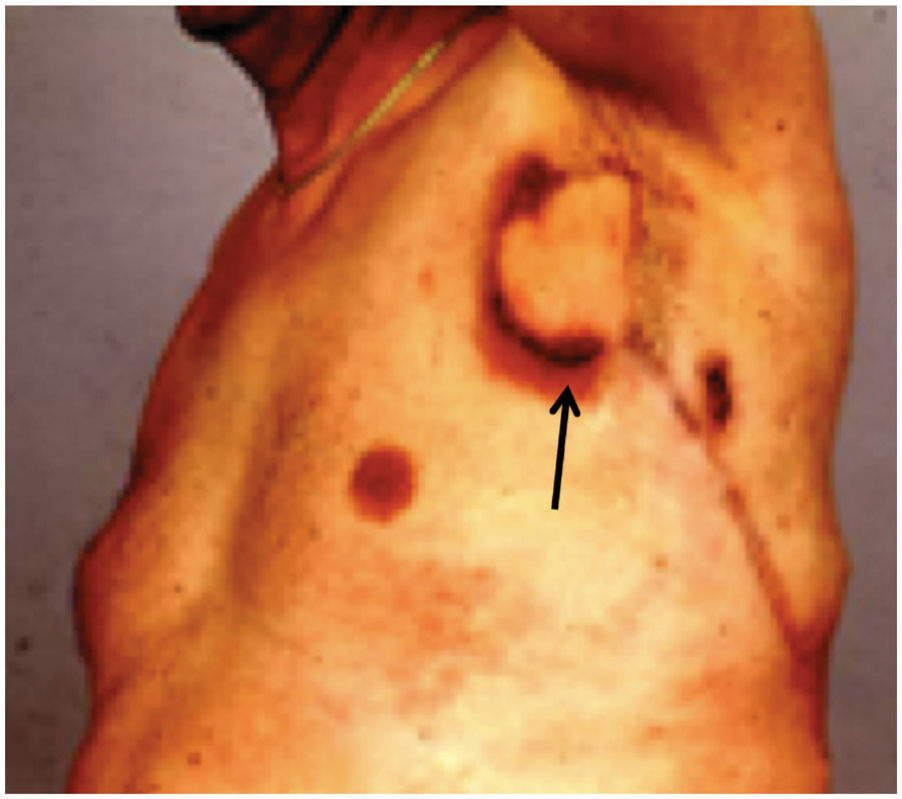
The fistula recurred (arrow) despite the transferred LD-flap.

Postoperatively the leakage stopped, but after a few months new fistulas arose in the edges of the flap, and leakage of lymph resumed (Figure 3).

In June 1995, two drains were placed after excision of the fistulas in order to aid in the healing process. However, the drains were blocked and were removed.

**Figure 4.**
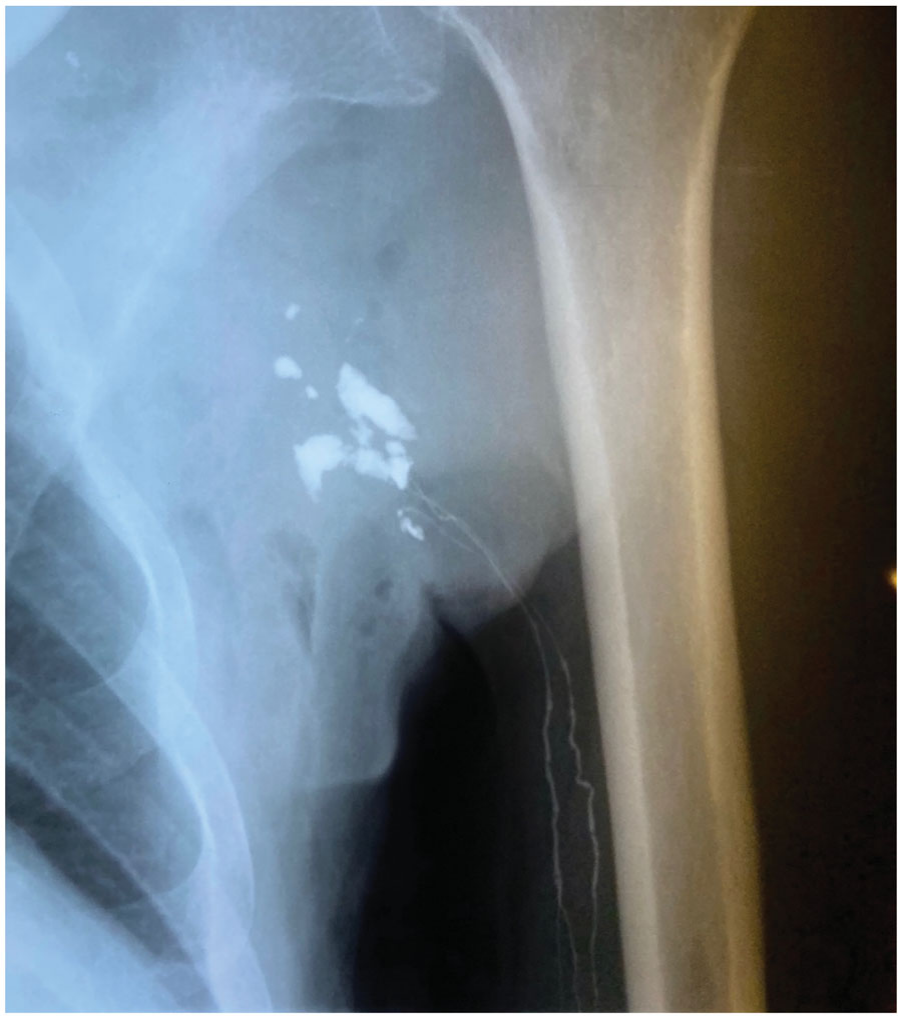
Lymphangiography shows lymph vessels emptying into multiple cavities in the axilla and the lateral thoracic wall.

In December 1995, a lymphangiography was performed, which showed vessels of normal caliber in the left arm emptying into multiple cavities in the axilla and the lateral thoracic wall. No further transport of lymph above the axillary region was detected (Figure 4).

In January 1997, our microvascular team, supervised by Professor Rüdiger Baumeister, Department of Micro, Hand and Reconstructive Surgery, Ludwig-Maximilians-University of Munich, Germany transplanted lymph collector vessels in order to normalize the lymphatic flow.

**Figure 5.**
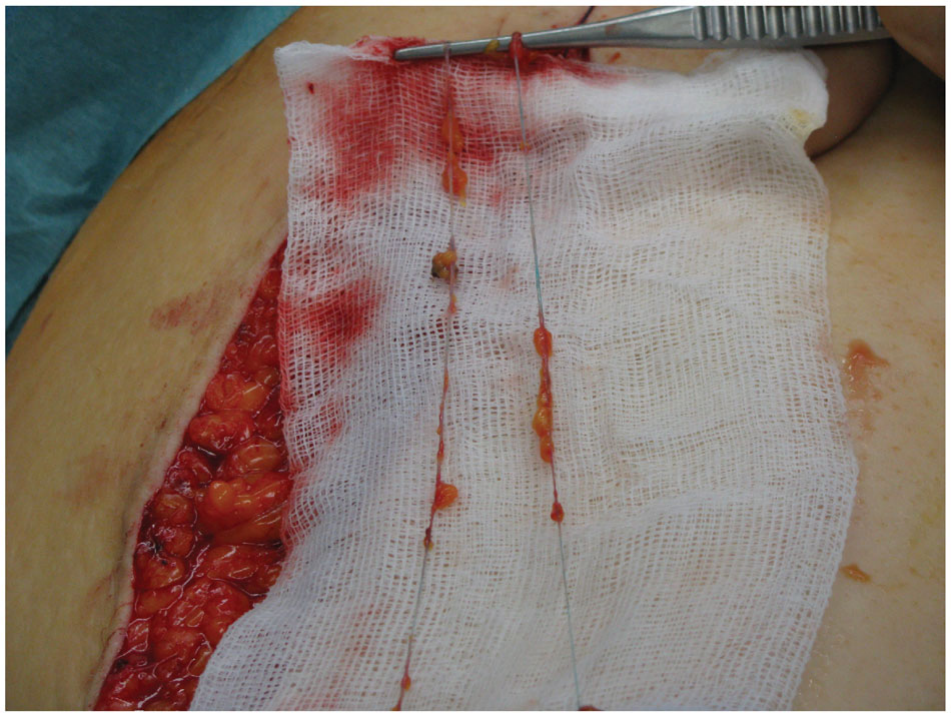
Two lymphatic vessels from the thigh are ready for transplantation.

Before harvesting lymphatic vessel grafts from the thigh, lymphoscintigraphy of the leg was performed, showing normal lymph flow, which is a prerequisite for harvest [11]. Two superficial main lymphatic collectors, each 23 cm in length, together with three branches were harvested from the inner aspect of the thigh. The axillary border of the LD-flap was opened. Patent Blue V^®^ (Guerbet, GmbH) was interdigitally injected into the affected arm. Stained lymphatic vessels in the upper arm below the axilla were identified, centrally ligated in order to stop the influx into the fistula, and transected. The lymphatic collectors were then ready for anastomosis with the lymphatic grafts (Figure 5).

In the neck region, lymph vessels draining lymph toward the left venous angle were identified to receive the lymph fluid from the arm. Temporarily, a moistened silicon tube was placed within the subcutaneous tissue between the two incisions. The grafts could then be pulled without friction from the upper arm towards the neck. After removal of the tube, the grafts were anastomosed without tension to the ascending lymphatic main collectors at the upper arm. In the neck, the central endings of the grafts were anastomosed to lymphatic collectors running towards the left venous angle. The end-to-end anastomoses were created with single stitches in the so-called tension-free technique [4]. using Vicryl 10-0 (Ethicon^®^, USA) and a BV 75-4 needle. With this procedure, the area of the axilla and the origin of the fistula were bypassed and the influx into the fistula was impeded while securing the lymphatic transport from the arm.

During the first postoperative year, spontaneous emptying of lymph occurred occasionally and ceased over time. The patient’s pain disappeared and the range of motion in the shoulder normalized. Over the following years, only small leaks were noted in conjunction with an upper respiratory tract viral infection, and these ceased after compression. The patient also had four episodes of erysipelas in the area, which were successfully treated with antibiotics. No leakage has occurred since 2003.

**Figure 6.**
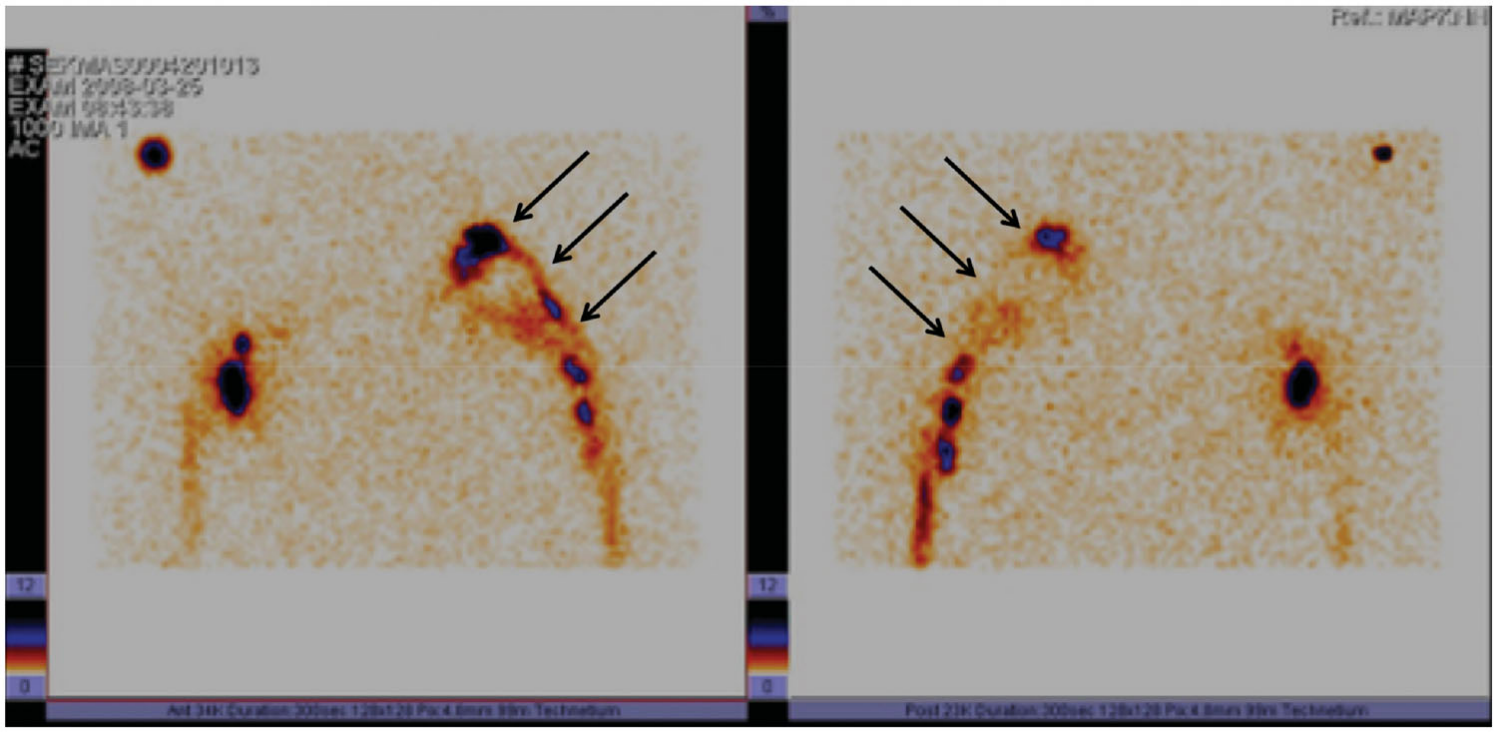
Lymphoscintigraphy, performed 11 years after grafting clearly shows lymph flow along the route of patent lymphatic grafts from the left arm to the neck (arrows). Left, frontal view; right, dorsal view.

In 2008, 11 years after the grafting, a lymphoscintigraphy showed good bilateral flow within the arms. On the affected side the flow of lymph was bypassing the axillary region *via* the lymphatic grafts whereas on the contralateral arm the lymph nodes in the axilla were depicted. (Figure 6).

**Figure 7.**
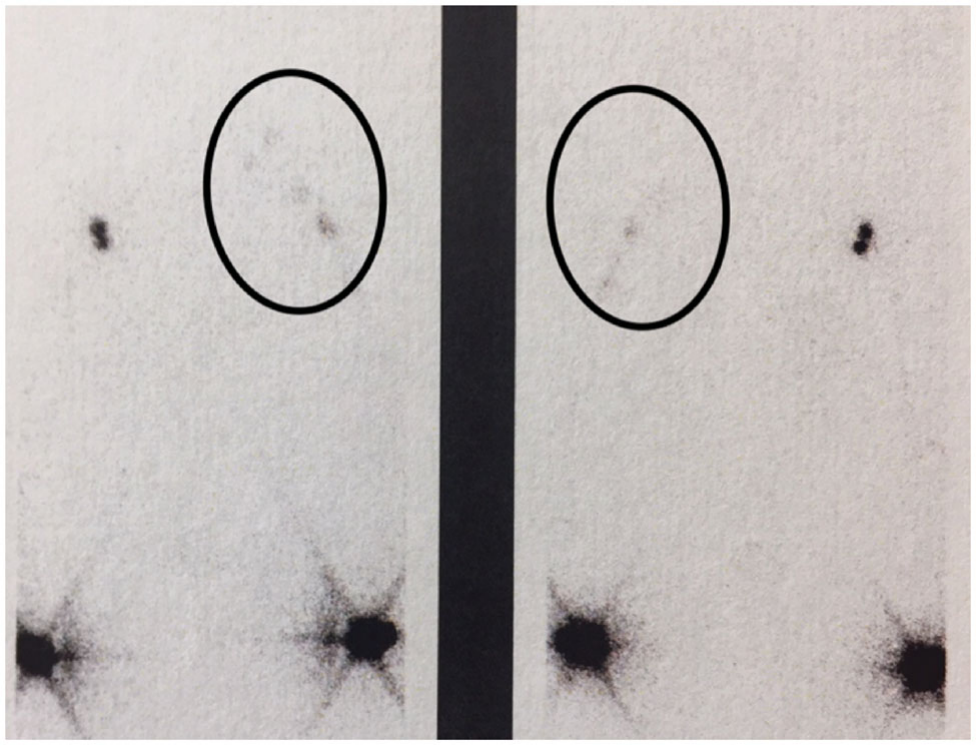
Outcome after 23 years showing transport of lymph from both arms in a lymphoscintigraphy. Ovals encircle the flow in the left side where lymph is transported *via* the grafts to of the neck. Left, frontal view; right, dorsal view.

In 2017, 23 years after the lymph vessel transplantation, again a similar pattern of activity was seen in a lymphoscintigraphic control study on both arms, indicating again lymph flow along with the patent lymphatic grafts to the neck (Figure 7).

## Discussion

In our patient different surgical interventions in the axilla took place in a local hospital following a minor wound on the index finger and bursitis. The major problem in the diabetic patient arose in the axilla with fluid accumulation and signs of massive tenderness compressing the brachial plexus. The origin of a hematoma remained unclear as well as the onset of a palpable mass showing an inflammatory reaction and focal fat necrosis. Together with the removal of an enlarged lymph node, it must be stated that obviously the whole content of the axilla was involved. This resembles the status of total axillary revision and radiation in an oncologic situation with a high incidence of secondary lymphedema.

Despite the absence of lymphatic tissue in a first specimen later on a pseudocapsule with dilated lymphatic vessels within an abscess formation was described by the pathologists. This means that within an infected area lymph came out from dilated lymphatic vessels and filled the axilla, resembling a lymphocele. This is clearly seen in Figure 4.

Altogether, a blockade at a narrowing of the lymphatic outflow from the arm was created. The lymphatic transport capacity was reduced to almost zero whereas the lymphatic load was not changed or, in the case of an infection, increased.

With lymphatic collectors open to the surface, lymphedema did not develop as long as the lymphorrhea persisted.

Attempts of treating the lymphatic fistula have been focused on closing it by identifying the leakage, ligation, diathermy, compression, and finally covered by an LD-flap.

However, the blocked lymphatic transport, which is the underlying problem, can be solved in a natural way by restoring the lymphatic pathway. The lymphatic transport capacity will be improved or even normalized by increasing the number of functioning lymphatic collectors [3–5,7–10]. There is proven evidence of increased lymphatic transport up to normal after bypassing the axilla with autogenous lymphatic collector grafts [11–13] as shown by nuclear medical measurements according to Kleinhans [6].

In our patient, the postoperative lymphatic flow was demonstrated along the route of the lymphatic grafts. On the contralateral arm, the axillary lymph nodes were depicted at the axilla as usual in normal lymphoscintigraphies, whereas on the affected side activity was depicted higher up until the neck where anastomoses between the grafts and lymphatic vessels have been performed.

The lymphatic collectors in the arm have been pumping lymph out of the fistula for years and were also able to pump lymph into the lymphatic grafts.

This is of specific benefit in this case. However, lymphatic grafts can also be used in longstanding lymphedemas where the function of the lymphatic collectors within the edematous tissue is poor because of their fibrosed walls. This possibility was also reported by a group performing different types of microsurgical procedures to treat lymphedema [14,15]. The reason for this may be that lymphatic collectors remain active after removing them from the body [16]. Therefore, the grafted lymphatic collectors can act as suction pumps emptying the residual lumen of the fibrosed lymphatic collectors in cases of longstanding lymphedema, and be of additional help like in our patient.

Experimental data indicate that simple venous interposition would be less promising, as they may act just as simple tubes [17].

Other possibilities would theoretically be to perform lymphatic to venous anastomoses [18–21]. Their effect, however, is dependent on a persistently higher pressure within the lymphatic system compared to the venous system. In our patient, the development of lymphedema would have been necessary prior to create such connections. In fact, the fistula reopened in our patient before lymphedema developed.

Using lymphatic collector grafts to connect lymphatic vessels in front and behind the area of blockade of the lymphatic system respects the low pressure within the lymphatic vascular system. Furthermore, only lymphatic fluid is present within the lymph-to-lymph anastomosis. This avoids the higher rate of thrombotic closure in lymphatico-venous shunting procedures if blood contacts lymph [22].

Another possibility to reduce the irritation of the anastomotic areas is the use of absorbable suture material instead of non-absorbable material. The foreign body reaction has been shown to be minimized and to disappear after weeks [23].

The implantation of free microsurgically transferred lymph nodes was contraindicated because of the contaminated wound in the axilla [24]. Furthermore, the influx into the lymph nodes by a spontaneously, newly established afferent capillary lymph to lymph connection would have been needed and establishing efferent lymphatic outflow would have been difficult.

Long-term open vessels are necessary for a persistent effect of vascular surgical procedures. This long-term patency has already been shown in autogenous lymphatic grafts for more than 7 years [10]. With this patient, we demonstrated patency for more than 20 years.

**Figure 8.**
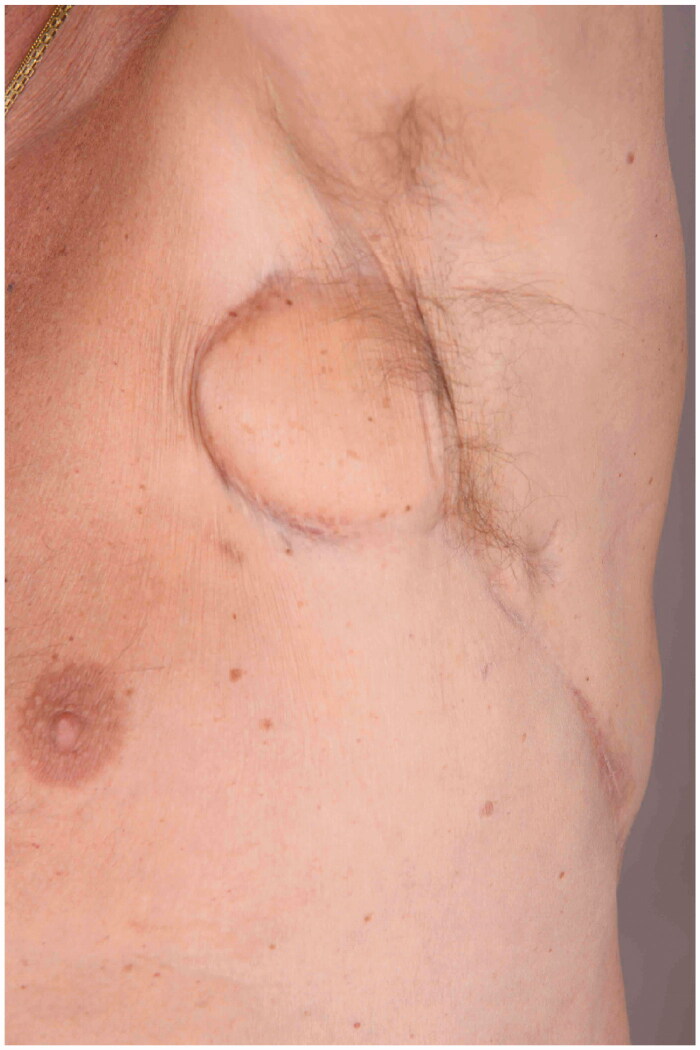
Normal status after 24 years without leakage.

In conclusion, this case demonstrates successful transplantation of lymph vessels in a patient with chronic lymphorrhea in the axilla. Open and functioning grafts have been demonstrated after more than 20 years by lymphoscintigraphy (Figure 8). Thus, the results have remained stable up to March 2021.
